# On the Role of English as a Foreign Language Learners’ Individual Differences in Their Use of Grammar Learning Strategies

**DOI:** 10.3389/fpsyg.2022.853158

**Published:** 2022-03-03

**Authors:** Shaoru Li

**Affiliations:** School of Journalism and Communication, Shandong University, Jinan, China

**Keywords:** GLSs, EFL learners, individual differences, desire to learn a second language, motivation, willingness to communicate

## Abstract

As the use of strategies facilitates the tedious process of language learning, a multitude of studies have been conducted on language learning strategies and their educational consequences. Nonetheless, grammar learning strategies (GLSs) have not been widely studied. Moreover, no review study has been carried out to illustrate the role of individual differences in the use of GLSs. To address the existing gaps, the present review study intends to explain the role of English as a foreign language (EFL) learners’ individual differences (i.e., desire to learn a second language, motivation, and willingness to communicate) in their employment of GLSs. The favorable impact of individual difference variables on grammar learning strategy use was proved using the theoretical and empirical evidence. Future research directions and pedagogical implications are also discussed.

## Introduction

Language learning strategies employed by second language learners in classroom contexts facilitate the learning process and make language learning more efficient, delightful, and enjoyable (Oxford et al., 2007). Because of this, a great deal of attention has been dedicated to language learning strategies, their definitions, and their subsets (e.g., Uslu et al., 2016; Feng et al., 2020; Pawlak, 2021a, to cite a few). However, research on grammar learning strategies (GLSs) is still in its infancy (Pawlak, 2012, 2020). Put simply, only a few investigations have been conducted into strategies of learning grammar. As put forward by Oxford and Amerstorfer (2018, p. 244), GLSs are “teachable, dynamic thoughts and behaviors that language learners consciously select and employ in specific contexts to improve their self-regulated, autonomous L2 grammar development for effective task performance and long-term efficiency”. To underline the importance of GLSs, De Keyser (2017) maintained that the efficient use of grammar strategies contributes to the growth of both explicit and implicit knowledge of grammar structures. In this regard, Pawlak (2018) also stated that those students who skillfully utilize various strategies to learn grammar are able to readily comprehend and remember the grammatical rules and structures. Additionally, Alsied et al. (2018) submitted that the efficient employment of GLSs will contribute to students’ higher grammatical competence and better linguistic performance. Similarly, Yeh (2021) also proposed that the use of GLSs will result in increased language achievement. Accordingly, identifying factors that may encourage L2 learners to employ GLSs throughout the learning process appears to be essential. To respond to this necessity, some researchers have delved into a variety of variables that may significantly contribute to the use of strategies in acquiring grammatical rules and structures (e.g., Rahimi et al., 2008; Abri et al., 2017; Zhou, 2017; Oxford and Amerstorfer, 2018; Zarrinabadi et al., 2021). Yet, the role of individual difference variables in the use of GLSs has remained elusive (Jabbari and Golkar, 2014; Khodadad and Kaur, 2018; Mistar and Zuhairi, 2020).

Individual differences generally refer to “dimensions of enduring personal characteristics that are assumed to apply to everybody and on which people differ by degree” (Dörnyei, 2006, p. 42). Individual differences in the educational domain pertain to a set of characteristics that mark pupils as unique and distinct learners (Winke, 2007; Hallett et al., 2012). As noted by Ellis (2004, p. 525), “*personality, language aptitude, desire to learn a second language, willingness to communicate, motivation, learning strategies,* and *learning styles*” are the main domains of individual differences among language learners. Among them, desire to learn a second language, motivation, and willingness to communicate were opted to be the main focus of this review study. As Dörnyei and Ryan (2015) mentioned, individual differences can drastically influence different aspects of language learning. Similarly, Griffiths and Soruç (2020) also submitted that individual differences are the significant predictors of students’ language achievement.

Due to the significance of individual differences in learning a new language (Dörnyei and Ryan, 2015; Griffiths and Soruç, 2020), a plethora of studies have been conducted to explore the educational consequences of language learners’ individual differences (e.g., Ardasheva and Tretter, 2013; Fillmore et al., 2014; Jaeggi et al., 2014; Grey et al., 2015; MacIntyre et al., 2016; Pawlak et al., 2016; Pawlak, 2017a,b). Notwithstanding, scant attention has been dedicated to the role of language learners’ individual differences in their use of GLSs (Jabbari and Golkar, 2014; Khodadad and Kaur, 2018; Mistar and Zuhairi, 2020). Moreover, to the best of the author’s knowledge, no review study has been done to offer various definitions of these variables (i.e., individual differences and grammar learning strategies) and illustrate their interactions in light of the theoretical and empirical evidence. This review study seeks to narrow this gap by illustrating the role of EFL learners’ individual differences in their use of GLSs.

### Individual Differences

Individual differences generally refer to “dimensions of enduring personal characteristics that are assumed to apply to everybody and on which people differ by degree” (Dörnyei, 2006, p. 42). Individual difference variables have been broadly classified as *affective*, *cognitive*, and *personality-related* factors (Jonassen and Grabowski, 2012; Kormos, 2012). The first group of individual difference variables (i.e., affective factors), which is the main focus of this review study, encompasses various emotional traits such as motivation, desire to learn, and willingness to communicate. In the language education domain, motivation explains why students initiate the language learning process, how long they are willing to stick with it, and how much effort they put into it (Winne and Hadwin, 2008; Dörnyei, 2010). Put differently, motivation provides “the primary impetus to initiate L2 learning and later the driving force to sustain the long, often tedious process of language learning” (Dörnyei and Ryan, 2015, p. 72). Language learners’ desire to learn as another emotional trait deals with students’ strong feelings for acquiring a new language (Willey and Gardner, 2009). Finally, students’ willingness to communicate pertains to “their readiness to enter into discourse at a particular time with a specific person or persons, using an L2” (MacIntyre et al., 1998, p. 548).

There is a multitude of studies to demonstrate that students’ individual differences substantially influence their academic behaviors and learning outcomes (e.g., Ardasheva and Tretter, 2013; Fillmore et al., 2014; Grey et al., 2015; Pawlak, 2017a). In their study, Ardasheva and Tretter (2013), for instance, found a strong association between EFL students’ individual differences and their language achievement. Further, Fillmore et al. (2014) reported a close connection between language learners’ individual differences and their language ability. In a similar vein, Grey et al. (2015) discovered that individual differences can remarkably affect language learners’ academic development. Additionally, Pawlak (2017a) found individual difference variables to be the significant determinants of students’ academic success.

### Grammar Learning Strategies

Language learning strategies generally pertain to “actions and thoughts that learners consciously employ to make language learning and/or language use easier, more effective, more efficient, and more enjoyable” (Pawlak, 2009, p. 44). Likewise, GLSs as a type of language learning strategies refers to a set of adaptable thoughts and actions that L2 learners deliberately adopt to improve their grammatical competence and task performance (Pawlak, 2008, 2013a,b; Oxford et al., 2018). Like other types of language learning strategies, GLSs possess some unique features (Takeuchi et al., 2007): (a) GLSs are the actions that students perform, indicating that they are taking an active role in the learning process; (b) their employment is intentional; (c) they are entirely optional; (d) their usage necessitates focused, goal-oriented action; (e) they are employed to keep the learning process under control; and (f) they are designed to make the learning process easier. Various attempts have been made to offer a detailed and comprehensive classification of GLSs (e.g., Briewin et al., 2013; Chen, 2016; Comajoan, 2019). Chen (2016), for instance, grouped different grammar learning strategies into four main categories: “*Cognitive strategies*,” “*metacognitive strategies*,” “*social strategies*,” and “*affective strategies*” (Table 1). Retrieved from Chen (2016, p. 618).

**Table 1:** Classification of grammar learning strategies.

Category	Strategies
Cognitive Strategies	“Remembering grammar by generating recalled images” “Generalizing the grammar rules”
Metacognitive Strategies	“Making plans for learning grammar” “Checking the outcomes of learning grammar”
Social Strategies	“Applying the learned rules in communication” “Exchanging feedback in a language activities”
Affective Strategies	“Having an active state of mind in grammar learning” “Having a feeling of assurance in grammar learning”

To date, several empirical studies have examined the relationship between grammar learning strategy use and learning outcomes (e.g., Salahshour et al., 2013; Khatib and Ruhi Athar, 2015; Zekrati, 2017; Supakorn et al., 2018; Amroune and Charik, 2020; Azizmohammadi and Barjesteh, 2020; Nuraini, 2020). Zekrati (2017), for instance, explored the association between grammar learning strategy use and language attainment among Iranian EFL learners. To do this, 300 Iranian EFL learners with different language proficiency levels took part in this study. To gather the required information, the “*Oxford Solution Test*” and the “*Grammar Learning Strategies Questionnaire*” were distributed among participants. Performing Pearson correlation coefficient, the researcher found a strong and favorable connection between Iranian EFL learners’ use of grammar learning strategies and their language attainment. By the same token, Azizmohammadi and Barjesteh (2020) investigated the connection between Iranian EFL students’ use of grammar learning strategies and their grammar performance. In doing so, 80 Iranian EFL learners were invited to fill out the grammar learning strategies questionnaire (Oxford, 1990). They were also asked to respond to a grammar test. The results demonstrated a substantial association between EFL students’ use of grammar learning strategies and their performance on the grammar test. In a similar vein, Amroune and Charik (2020) studied language learners’ use of grammar learning strategies in relation to their grammar competence. To this end, 62 university students volunteered to participate in this study. Some semi-structured interviews were performed to assess students’ tendency to employ grammar learning strategies. Moreover, a grammar test was given to participants to evaluate their grammar competence. The findings suggest that students’ use of grammar learning strategies is positively associated with their grammatical competence.

### The Role of EFL Learners’ Individual Differences in Their Use of GLSs

Concerning the role of EFL students’ individual differences in their use of GLSs, Pawlak (2021b) submitted that individual differences, such as desire to learn, motivation, and willingness to communicate, can largely influence grammar learning strategy use. That is, students with a higher degree of motivation, academic desire, and willingness to communicate are more likely to employ strategies for mastering grammatical rules and structures. Similarly, Oxford and Amerstorfer (2018) argued that the employment of language learning strategies, notably GLSs, may be remarkably affected by individual learner characteristics (e.g., motivation, attitude, interest, and willingness to communicate). To them, highly motivated learners are more inclined to make use of language learning strategies. They also stated that those students who have positive beliefs and attitudes about learning a new language tend to choose and utilize efficient strategies in the learning process.

## Empirical Studies

Despite the importance of individual difference variables, such as motivation, academic desire, attitude, and willingness to communicate in grammar learning strategy use (Oxford and Amerstorfer, 2018; Pawlak, 2021b), limited attention has been paid to the role of these variables in EFL students’ employment of GLSs. That is, the influence of individual differences on the use of GLSs has remained marginal (Pawlak and Oxford, 2018). Put simply, a limited number of investigations have been performed in this regard (Jabbari and Golkar, 2014; Khodadad and Kaur, 2018; Mistar and Zuhairi, 2020). For instance, Jabbari and Golkar (2014) delved into the role of attitude as an individual difference variable in EFL students’ use of GLSs. To do so, the “*Strategy Inventory for Language Learning*” and the “*Attitude/Motivation Test Battery*” were administered to 100 EFL students. The results evinced that those EFL students who possess positive beliefs and attitudes toward English language grammar are better users of GLSs. In a same vein, Khodadad and Kaur (2018) examined the role of academic motivation as another instance of individual difference variables in grammar learning strategy use. To this end, two valid questionnaires were distributed among 240 Iranian EFL students. Relying on the participants’ answers, the researchers reported that grammar learning strategy use can be dramatically affected by students’ level of motivation. That is, students with higher levels of motivation are more likely to utilize GLSs. More recently, Mistar and Zuhairi (2020) studied several individual difference variables in relation to GLSs. They attempted to figure out the interrelationship among motivation, aptitude, personality, and grammar learning strategy use. To this aim, a set of questionnaires were administered to 280 EFL students. Inspecting the correlation of questionnaires, they found that only students’ motivation can significantly affect their use of grammar strategies.

## Conclusion, Implications, and Suggestions for Future Research

So far, the existing definitions, characterizations, and classifications of individual differences and GLSs were outlined. With respect to the findings of previous studies, the role of EFL students’ individual differences in their use of GLSs was illustrated as well. In light of the empirical evidence, one can conclude that individual difference variables, such as motivation, academic desire, and willingness to communicate, may favorably affect both the quality and quantity of grammar learning strategy use. That is, EFL students with high levels of motivation, academic desire, and willingness to communicate are better users of GLSs (Oxford and Amerstorfer, 2018; Pawlak, 2021b).

This finding appears to be instructive for English language teachers and teacher trainers. To enhance EFL students’ use of GLSs, teachers are required to improve their pupils’ positive emotional traits, including motivation, desire to learn, and willingness to communicate. Given the undeniable role of students’ motivation, desire to learn, and willingness to communicate in their use of GLSs, teacher trainers are expected to educate their trainees how to develop these positive attributes in their students. As the review of previous studies revealed, only a few investigations (Jabbari and Golkar, 2014; Khodadad and Kaur, 2018; Mistar and Zuhairi, 2020) have been conducted to delve into the role of students’ individual differences in their use of grammar strategies. Thus, further investigation into this topic is strongly recommended.

## Data Availability Statement

The original contributions presented in the study are included in the article/supplementary material, and further inquiries can be directed to the corresponding author.

## Ethics Statement

The studies involving human participants were reviewed and approved by the Shandong University Academic Ethics Committee. The patients/participants provided their written informed consent to participate in this study.

## Author Contributions

The author confirms being the sole contributor of this work and has approved it for publication.

## Conflict of Interest

The author declares that the research was conducted in the absence of any commercial or financial relationships that could be construed as a potential conflict of interest.

## Publisher’s Note

All claims expressed in this article are solely those of the authors and do not necessarily represent those of their affiliated organizations, or those of the publisher, the editors and the reviewers. Any product that may be evaluated in this article, or claim that may be made by its manufacturer, is not guaranteed or endorsed by the publisher.
